# Comparative analysis of treatment response to PSMA radioligand therapy using Ga-68 PSMA-11 and F-18 DCFPyL PET imaging

**DOI:** 10.3389/fonc.2026.1932583

**Published:** 2026-10-02

**Authors:** Chae Moon Hong, Abuzar Moradi Tuchayi, Mansour Abtahi, Stellamaris Nwihim, Surekha Yadav, Robert R. Flavell, Thomas A. Hope

**Affiliations:** 1 Department of Radiology and Biomedical Imaging, University of California, San Francisco, San Francisco, CA, United States; 2 Department of Nuclear Medicine, Kyungpook National University, Daegu, Republic of Korea; 3 Helen Diller Family Comprehensive Cancer Center, University of California, San Francisco, San Francisco, CA, United States

**Keywords:** DCFPyL, PET, prostate cancer, PSMA, PSMA-11, radioligand therapy

## Abstract

**Purpose:**

Multiple prostate-specific membrane antigen (PSMA) PET tracers are used for patient selection in radioligand therapy (RLT). This study compared treatment response to RLT across different PSMA tracers.

**Methods:**

We retrospectively analyzed patients who underwent Lu-177 PSMA-617 RLT with baseline imaging using either Ga-68 PSMA-11 or F-18 DCFPyL PET. PET parameters were measured, including SUV_mean_, SUV_max_, and total tumor volume (TTV). Univariable and multivariable logistic regression analyses were performed to evaluate factors associated with PSA50 response, and Cox proportional hazards regression analyses were performed to evaluate factors associated with overall survival.

**Results:**

Of 140 patients analyzed (Ga-68 PSMA-11: n=51; F-18 DCFPyL: n=89), SUV_max_ and SUV_mean_ did not differ significantly between groups; however, TTV was significantly higher in the Ga-68 PSMA-11 group (p=0.038). In multivariable analysis adjusted for age, baseline PSA, PET tracer type, SUVmean, and log-transformed TTV, PET tracer type was not significantly associated with PSA50 response (OR, 1.318; 95% CI, 0.585–2.970; p=0.505), whereas higher SUVmean was associated with greater odds of PSA50 response (OR, 1.285; 95% CI, 1.126–1.466; p<0.001). Overall survival did not differ significantly between tracer groups (HR, 0.760; 95% CI, 0.507–1.138; p=0.183), with median overall survival of 8.4 and 12.6 months. In multivariable analysis, PET tracer type was not significantly associated with overall survival (HR, 0.736; 95% CI, 0.475–1.139; p=0.168), whereas higher SUVmean was associated with a lower risk of death (HR, 0.886; 95% CI, 0.825–0.950; p<0.001) and higher log-transformed TTV was associated with a higher risk of death (HR, 1.415; 95% CI, 1.194–1.678; p<0.001).

**Conclusion:**

PET tracer type was not significantly associated with PSA50 response or overall survival following RLT. Higher SUV_mean_ was associated with greater odds of PSA50 response and a lower risk of death, whereas higher TTV was associated with overall survival.

## Introduction

Metastatic castration-resistant prostate cancer (mCRPC) represents a significant clinical challenge, even with the advent of multiple novel therapeutic agents. Radioligand therapy (RLT) using Lu-177 PSMA-617 (lutetium Lu 177 vipivotide tetraxetan) has emerged as an effective treatment option, demonstrating improved overall survival in the VISION and PSMAfore trials (1, 2). In both trials, Ga-68 PSMA-11 (gozetotide) was used for patient selection.

Three PSMA PET radiopharmaceuticals have been evaluated and approved by the FDA at initial staging and biochemical recurrence: Ga-68 PSMA-11 (gozetotide), F-18 DCFPyL (piflufolostat), and F-18 rhPSMA-7.3 (posluma) (3–5). All approved agents are generally considered equivalent (6), and the choice imaging agent is largely dictated by institutional availability and reimbursement.

Regarding patient selection for RLT, both pivotal clinical trials used Ga-68 PSMA-11 for patient selection (1, 7), and it remains the reference tracer for RLT eligibility assessment. Although pharmacokinetic and biodistribution profiles differ across PSMA-targeted tracers, current guidelines recommend evaluating the pattern of PSMA expression irrespective of the specific tracer used (8). We previously reported that patients selected using F-18 DCFPyL achieved a superior PSA50 response rate compared to those selected using Ga-68 PSMA-11 (9), which was unexpected.

We therefore compared the outcomes of patients selected using either Ga-68 PSMA-11 or F-18 DCFPyL in a larger population of patients to validate our prior results and evaluated whether quantitative PET parameters could explain the previously observed difference in treatment response.

## Materials and methods

### Patient population

We retrospectively identified 220 patients who underwent baseline PSMA PET before the first cycle of Lu-177 PSMA-617 RLT between May 2019 and February 2025 at our institution. Of these, 80 patients had been included in our previously published study (9) and were excluded from the primary analysis to allow evaluation of the previously reported findings in an independent cohort. The remaining 140 patients constituted the primary cohort of the current study, including 51 patients imaged with Ga-68 PSMA-11 and 89 imaged with F-18 DCFPyL. No additional systemic anticancer treatment other than ADT or ARPI therapy was administered between baseline PSMA PET and the first RLT administration. The previously published cohort of 80 patients did not overlap with the 140-patient primary cohort and was analyzed separately as a secondary analysis (**Figure 1**).

**Figure 1:**
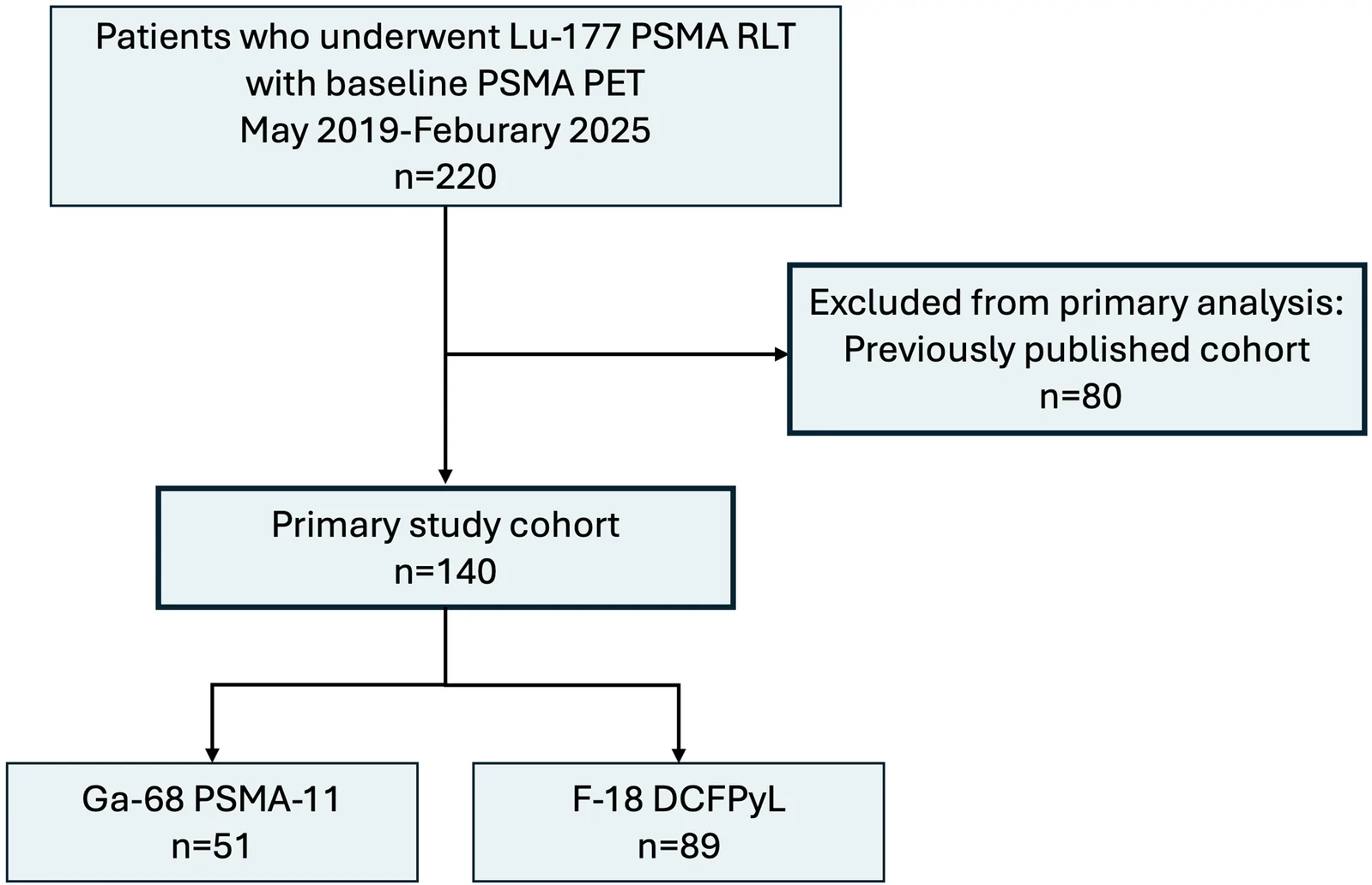
Study flow diagram showing the derivation of the primary study cohort.

### PSMA PET and quantitative image analysis

PET imaging protocol was based on standard of care and was not prospectively defined (6). Tumor segmentation was performed using predefined standardized uptake value (SUV) thresholds. An initial SUV threshold of ≥3.0 was applied for lesion segmentation. Threshold modification was performed on a lesion-by-lesion basis. If an individual suspected metastatic lesion was not adequately delineated using the initial threshold, the threshold was reduced in 0.5-unit increments, to a minimum SUV threshold of 1.0, until the visually identified extent of the lesion was adequately encompassed by the segmentation contour. For liver metastases, normal liver SUV_mean_ was measured using a 3-cm-diameter circular region of interest placed in normal liver parenchyma at the inferior portion of right hepatic lobe, avoiding focal lesions, and a threshold of 1.5 × the mean SUV of normal liver was used. All segmentation results were reviewed by a nuclear medicine physician, and manual correction was performed as necessary. Physiologic PSMA uptake and urinary activity were manually excluded based on the nuclear medicine physician’s assessment. The reader was blinded to PET tracer type and clinical outcomes during segmentation. Image segmentation was performed using MIM software (version 7.4.4; MIM Software Inc., Cleveland, OH). Three whole-body parameters were calculated for each patient: SUV_max_, SUV_mean_, and total tumor volume (TTV).

### Response assessment

Prostate Specific Antigen (PSA) values were drawn on the day of each RLT treatment and between treatments. The maximum decline in PSA occurring at any time during RLT or within 12 weeks after completion was used for the PSA response analysis. A decline of 50% from baseline PSA was defined as PSA50 response. ADT and ARPI therapy were permitted during RLT; no additional systemic anticancer therapies were administered concomitantly with RLT. Overall survival was calculated from the date of the first RLT administration until death, with censoring at the date of last follow-up. Kaplan-Meier curves were generated for overall survival. Median follow-up duration was estimated using the reverse Kaplan–Meier method.

### Statistical analysis

Categorical variables were reported as frequencies and percentages. Continuous variables were summarized as medians with interquartile ranges (IQRs) for non-normally distributed data and as means with standard deviations (SDs) for normally distributed data. Categorical variables were compared using the chi-square test or Fisher’s exact test, as appropriate. Continuous variables were compared using the Student’s t test for normally distributed data and the Wilcoxon rank-sum test for nonparametric data.

Univariable and multivariable logistic regression analyses were performed to evaluate factors associated with PSA50 response, and odds ratios (ORs) were reported with 95% confidence intervals (CIs). Univariable and multivariable Cox proportional hazards regression analyses were performed to evaluate factors associated with overall survival, and hazard ratios (HRs) were reported with 95% CIs. Multivariable models included PET tracer type, SUV_mean_, TTV, age at first RLT, and baseline PSA. Because TTV showed a markedly right-skewed distribution, it was natural log-transformed for regression analyses. SUV_max_ and SUV_mean_ were correlated (Spearman ρ=0.723, p<0.001); therefore, SUV_mean_ was retained as the quantitative uptake parameter in the multivariable models. Statistical analyses were performed using R (version 4.5.1).

## Results

### Patient characteristics

Of the 220 patients who underwent Lu-177 PSMA-617 RLT, 80 patients from the previously published cohort were excluded from the primary analysis. The remaining 140 patients constituted the primary cohort, including 51 patients imaged with Ga-68 PSMA-11 and 89 imaged with F-18 DCFPyL. Prior systemic treatment history was available for 99 of 140 patients (70.7%) and was unavailable for 41 patients (29.3%), primarily because complete prior treatment records were unavailable for patients referred from outside institutions. The proportion of missing prior-treatment data did not differ significantly between tracer groups (p=0.580). Prior treatments were generally comparable between groups; however, other life-prolonging therapies were more frequent in the Ga-68 PSMA-11 group (68.4% vs. 44.3%, p=0.033). The median interval from baseline PSMA PET to the first RLT administration was 59.5 days (IQR, 38.75–85 days; range, 7–219 days). Sites of disease on PSMA PET were similar across tracers. SUV_max_ and SUV_mean_ did not differ significantly between groups. However, TTV was significantly higher in the Ga-68 PSMA-11 group compared with the F-18 DCFPyL group (p=0.038). Patient characteristics are summarized in **Table 1**.

**Table 1:** Patient characteristics.

Characteristic	Total (n=140)	Ga-68 PSMA-11 (n=51)	F-18 DCFPyL (n=89)	p-value
Age at first RLT (mean ± SD)	72.2 ± 8.2	69.9 ± 7.4	73.5 ± 8.4	0.009
Prior treatment				
Androgen deprivation treatment	96/99 (97.0%)	37/38 (97.4%)	59/61 (96.7%)	1.000
Androgen receptor pathway inhibitor	98/99 (99.0%)	38/38 (100.0%)	60/61 (98.4%)	1.000
Docetaxel	39/99 (39.4%)	17/38 (44.7%)	22/61 (36.1%)	0.518
Cabazitaxel	2/99 (2.0%)	2/38 (5.3%)	0/61 (0.0%)	0.145
Other life prolonging treatment	53/99 (53.5%)	26/38 (68.4%)	27/61 (44.3%)	0.033
Site of disease on PSMA PET				
Prostatic fossa	35/140 (25.0%)	14/51 (27.5%)	21/89 (23.6%)	0.761
Lymph nodes	90/140 (64.3%)	33/51 (64.7%)	57/89 (64.0%)	1.000
Bone	132/140 (94.3%)	48/51 (94.1%)	84/89 (94.4%)	1.000
Liver	31/140 (22.1%)	9/51 (17.6%)	22/89 (24.7%)	0.448
Lung	11/140 (7.9%)	2/51 (3.9%)	9/89 (10.1%)	0.327
Other Viscera	26/140 (18.6%)	12/51 (23.5%)	14/89 (15.7%)	0.360
SUV_max_ (median [IQR])	43.84 [28.47, 66.00]	43.61 [27.98, 66.23]	46.93 [29.37, 64.90]	0.834
SUV_mean_ (median [IQR])	7.61 [6.07, 10.03]	7.26 [6.23, 9.64]	7.75 [6.06, 10.22]	0.971
Total Tumor Volume (mL)	372.82 [138.39, 1050.20]	663.83 [178.03, 1540.24]	273.38 [110.20, 829.59]	0.038
Days from baseline PSMA PET to the 1st cycle (median [IQR])	59.50 [38.75, 85.00]	61.00 [35.00, 86.00]	57.00 [41.00, 82.00]	0.726
Number of Cycles (median [IQR])	3 [2, 4]	3 [2, 4]	3 [2, 5]	0.253
Baseline PSA (median, ng/mL [IQR])	127.14 [31.91, 311.85]	98.96 [26.34, 240.17]	133.08 [35.52, 331.37]	0.488
PSA50 Response rate	76/139 (54.7%)	26/51 (51.0%)	50/88 (56.8%)	0.624

### PSA50 response

PSA50 response was evaluable in 139 of 140 patients; one patient in the F-18 DCFPyL group had a baseline PSA level below the limit of detection and therefore could not be evaluated for PSA50 response. The PSA50 response rate was 51.0% (26/51) in the Ga-68 PSMA-11 group and 56.8% (50/88) in the F-18 DCFPyL group. The PSA waterfall plot demonstrated comparable PSA response patterns between the two tracer groups (**Figure 2**). PET tracer type was not significantly associated with PSA50 response in univariable analysis (OR, 1.265; 95% CI, 0.633–2.528; p=0.505). Higher SUVmax (OR 1.015; 95% CI 1.004–1.025; p=0.007) and SUVmean (OR 1.293; 95% CI 1.131–1.478; p<0.001) were significantly associated with PSA50 response, whereas log-transformed TTV was not (OR, 1.244; 95% CI, 0.964–1.606; p=0.093). In multivariable analysis adjusted for age, baseline PSA, PET tracer type, SUV_mean_, and log-transformed TTV, higher SUV_mean_ remained significantly associated with greater odds of PSA50 response (OR, 1.285; 95% CI, 1.126–1.466; p<0.001). PET tracer type (OR, 1.318; 95% CI, 0.585–2.970; p=0.505) and log-transformed TTV (OR, 1.357; 95% CI, 0.997–1.848; p=0.052) were not significantly associated with PSA50 response (**Table 2**).

**Figure 2:**
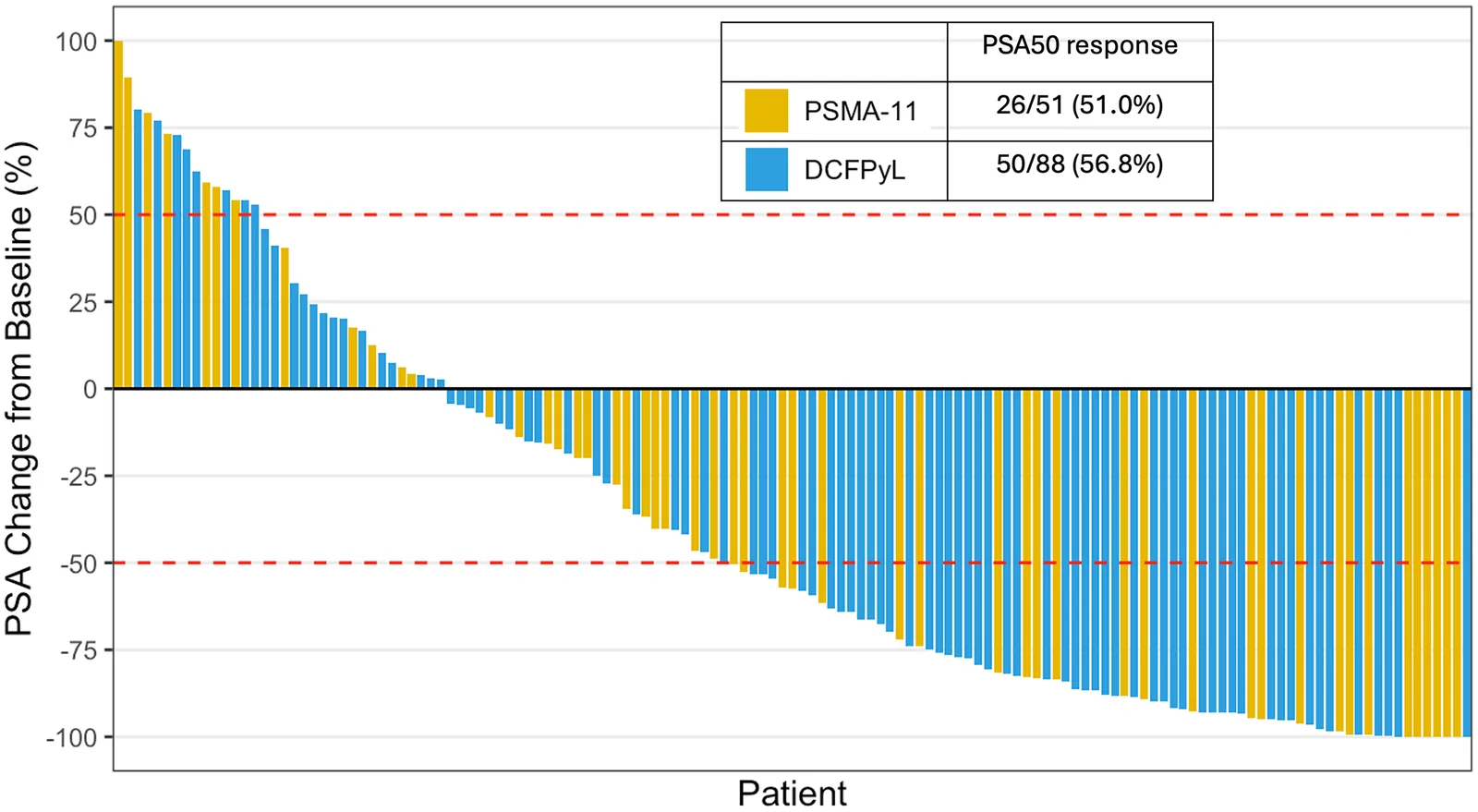
PSA waterfall plot by PET tracers.

**Table 2:** Univariable and multivariable analysis for PSA50 response and overall survival.

*Logistic regression analysis for PSA50 response*						
Variable	Univariable			Multivariable		
	OR	95% CI	*P value*	OR	95% CI	*P value*
*Age at first RLT*	1.031	0.988–1.077	0.158	1.038	0.988–1.090	0.136
*Baseline PSA*	1.000	0.999–1.000	0.536	1.000	0.999–1.000	0.476
*PET tracer (DCFPyL)*	1.265	0.633–2.528	0.505	1.318	0.585–2.970	0.505
*SUV_max_*	1.015	1.004–1.025	0.007			
*SUV_mean_*	1.293	1.131–1.478	<0.001	1.285	1.126–1.466	<0.001
*TTV* *(log-transformed)*	1.244	0.964–1.606	0.093	1.357	0.997–1.848	0.052

*Cox proportional hazard regression for overall survival*						
	Univariable			Multivariable		
	HR	95% CI	*P value*	HR	95% CI	*P value*
*Age at first RLT*	0.995	0.971–1.020	0.685	1.001	0.973–1.030	0.934
*Baseline PSA*	1.000	1.000–1.001	0.002	1.000	1.000–1.000	0.315
*PET tracer (DCFPyL)*	0.760	0.507–1.138	0.183	0.736	0.475–1.139	0.168
*SUV_max_*	0.992	0.987–0.998	0.006			
*SUV_mean_*	0.902	0.850–0.958	<0.001	0.886	0.825–0.950	<0.001
*TTV* *(log-transformed)*	1.431	1.209–1.694	<0.001	1.415	1.194–1.678	<0.001

### Overall survival

Among the 140 patients, 97 (69.3%) died during follow-up and 43 (30.7%) were censored at the date of last follow-up. The median follow-up duration was 22.6 months (95% CI, 18.7–25.5 months), estimated using the reverse Kaplan–Meier method. There was no significant difference in overall survival between the Ga-68 PSMA-11 and F-18 DCFPyL groups (HR 0.760, 95% CI 0.507–1.138, p=0.183), with median overall survival of 8.4 months and 12.6 months, respectively (**Figure 3**). In multivariable analysis adjusted for age, baseline PSA, PET tracer type, SUV_mean_, and log-transformed TTV, PET tracer type was not significantly associated with overall survival (HR, 0.736; 95% CI, 0.475–1.139; p=0.168). Higher SUV_mean_ was associated with a lower risk of death (HR, 0.886; 95% CI, 0.825–0.950; p<0.001), whereas higher log-transformed TTV was associated with a higher risk of death (HR, 1.415; 95% CI, 1.194–1.678; p<0.001) (**Table 2**).

**Figure 3:**
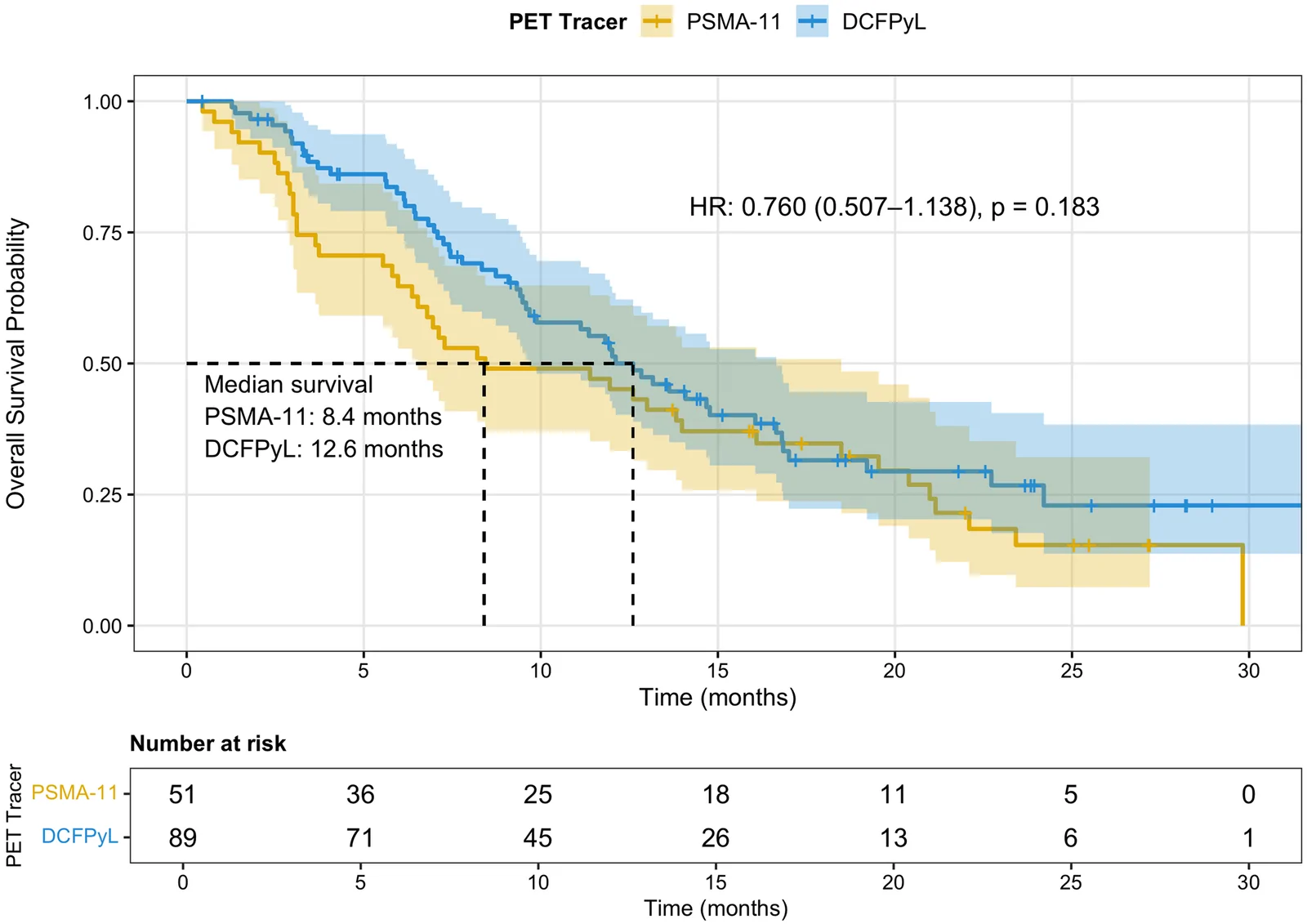
Overall survival by PET tracers.

### Additional analysis of previous study

In the previously published 80-patient cohort (Ga-68 PSMA-11, n=47; F-18 DCFPyL, n=33), PSA50 response rates were 43% (20/47) and 72% (24/33), respectively. In univariable analysis, F-18 DCFPyL was associated with greater odds of PSA50 response (OR, 3.049; 95% CI, 1.052–8.839; p=0.040). In multivariable analysis, PET tracer type was not significantly associated with PSA50 response (OR, 2.816; 95% CI, 0.901–8.800; p=0.075), whereas higher SUV_mean_ was significantly associated with greater odds of PSA50 response (OR, 1.210; 95% CI, 1.021–1.433; p=0.028). PET tracer type was not significantly associated with overall survival in multivariable analysis (HR, 0.850; 95% CI, 0.496–1.458; p=0.556). Detailed characteristics and regression analyses of the previously published cohort are provided in **Supplementary Tables 2**, **3**.

## Discussion

In contrast to our previous study, PET tracer type was not significantly associated with PSA50 response in the current independent cohort, including after adjustment for clinical and quantitative PET parameters. Higher SUV_mean_ was associated with greater odds of PSA50 response and a lower risk of death, whereas higher TTV was associated with a higher risk of death after adjustment for the included clinical and PET variables.

The absence of a significant difference in RLT outcomes between tracer groups after adjustment for PET parameters has important clinical implications. With the increasing complexity of baseline patient characteristics in the era of rapidly evolving systemic therapies for prostate cancer, prognostication has become more challenging. This heterogeneity makes comparisons across radiopharmaceuticals difficult. Our results suggest that adjusting for quantitative PET parameters, particularly SUV_mean_ and TTV, effectively accounts for this variability and may prevent misleading conclusions. These results support the incorporation of standardized PET parameter adjustment in future studies evaluating RLT outcomes. Furthermore, as new PSMA PET tracers continue to emerge beyond FDA-approved agents, our data indicate that quantitative PET parameters may be more critical than tracer selection itself.

Previous studies have demonstrated that higher PSMA SUV_mean_ was associated with greater biochemical response to RLT (7, 10). Consistent with these findings, higher SUV_mean_ was associated with greater odds of PSA50 response in our cohort after adjustment for the included clinical and PET variables. Higher SUV_mean_ was associated with improved overall survival in the extended follow-up analysis of the TheraP trial (7) and *post-hoc* analysis of VISION trial (11), although this association was not replicated in the Pros-TIC registry, a large real-world database (12). In our multivariable analysis, higher SUV_mean_ was associated with a lower risk of death after adjustment for the included clinical and PET variables. These findings support an association between higher PSMA uptake and favorable outcomes among patients receiving RLT.

Regarding overall survival, TTV derived from FDG PET has been previously shown to correlate with survival outcomes (7, 12). In the present study, higher PSMA PET-derived TTV was associated with a higher risk of death after adjustment for the clinical and PET variables included in the model, although the magnitude of this association varied over time. The recently proposed prostate cancer risk grouping by PSMA PET (PPP3) also incorporates PSMA PET-derived TTV as a prognostic parameter (13). These findings support an association between PSMA PET-derived tumor burden and survival outcomes in this patient population. Importantly, PSMA PET-derived and FDG PET-derived TTV represent tumor burden measured using different biological imaging targets and should not be considered interchangeable measures.

This study has several limitations. First, its retrospective, single-center design, nonrandom tracer allocation, baseline imbalances, and incomplete prior treatment history may have introduced selection bias and residual confounding. Although the revised multivariable analyses adjusted for age and baseline PSA, residual confounding from unmeasured or incompletely documented clinical factors cannot be excluded. Second, PSMA PET examinations were acquired across multiple scanners and institutions without prospectively standardized acquisition and reconstruction protocols. Tumor segmentation involved reader-dependent assessment and manual correction by a single nuclear medicine physician, and intra- and inter-reader reproducibility were not formally assessed. The interval between baseline PET and RLT also varied among patients; however, a sensitivity analysis restricted to patients who initiated RLT within 90 days yielded findings consistent with the primary analysis. Third, the use of the maximum PSA decline during RLT or within 12 weeks after completion may have introduced ascertainment bias. Finally, the tracer comparison was unpaired, and the study was not designed or powered to establish equivalence or noninferiority.

## Conclusion

In this retrospective cohort, PET tracer type was not significantly associated with PSA50 response or overall survival following RLT. Higher SUV_mean_ was associated with greater odds of PSA50 response and a lower risk of death, whereas higher TTV was associated with overall survival.

## Data Availability

The raw data supporting the conclusions of this article will be made available by the authors, without undue reservation.

## References

[1] Sartor O de Bono J Chi KN Fizazi K Herrmann K Rahbar K . Lutetium-177-PSMA-617 for metastatic castration-resistant prostate cancer. N Engl J Med. (2021) 385:1091–103. doi: 10.1056/NEJMoa2107322 34161051 PMC8446332

[2] Morris MJ Castellano D Herrmann K de Bono JS Shore ND Chi KN . 177Lu-PSMA-617 versus a change of androgen receptor pathway inhibitor therapy for taxane-naive patients with progressive metastatic castration-resistant prostate cancer (PSMAfore): a phase 3, randomised, controlled trial. Lancet. (2024) 404:1227–39. doi: 10.1016/S0140-6736(24)01653-2 39293462 PMC12121614

[3] Hope TA Eiber M Armstrong WR Juarez R Murthy V Lawhn-Heath C . Diagnostic accuracy of 68Ga-PSMA-11 PET for pelvic nodal metastasis detection prior to radical prostatectomy and pelvic lymph node dissection: A multicenter prospective phase 3 imaging trial. JAMA Oncol. (2021) 7:1635–42. doi: 10.1001/jamaoncol.2021.3771 34529005 PMC8446902

[4] Pienta KJ Gorin MA Rowe SP Carroll PR Pouliot F Probst S . A phase 2/3 prospective multicenter study of the diagnostic accuracy of prostate specific membrane antigen PET/CT with 18F-DCFPyL in prostate cancer patients (OSPREY). J Urol. (2021) 206:52–61. doi: 10.1097/JU.0000000000001698 33634707 PMC8556578

[5] Surasi DS Eiber M Maurer T Preston MA Helfand BT Josephson D . Diagnostic performance and safety of positron emission tomography with 18F-rhPSMA-7.3 in patients with newly diagnosed unfavourable intermediate- to very-high-risk prostate cancer: Results from a phase 3, prospective, multicentre study (LIGHTHOUSE). Eur Urol. (2023) 84:361–70. doi: 10.1016/j.eururo.2023.06.018 37414702

[6] Fendler WP Eiber M Beheshti M Bomanji J Calais J Ceci F . PSMA PET/CT: Joint EANM procedure guideline/SNMMI procedure standard for prostate cancer imaging 2.0. Eur J Nucl Med Mol Imaging. (2023) 50:1466–86. doi: 10.1007/s00259-022-06089-w 36604326 PMC10027805

[7] Hofman MS Emmett L Sandhu S Iravani A Buteau JP Joshua AM . Overall survival with [177Lu]Lu-PSMA-617 versus cabazitaxel in metastatic castration-resistant prostate cancer (TheraP): Secondary outcomes of a randomised, open-label, phase 2 trial. Lancet Oncol. (2024) 25:99–107. doi: 10.1016/S1470-2045(23)00529-6 38043558

[8] Kratochwil C Fendler WP Eiber M Hofman MS Emmett L Calais J . Joint EANM/SNMMI procedure guideline for the use of 177Lu-labeled PSMA-targeted radioligand-therapy (177Lu-PSMA-RLT). Eur J Nucl Med Mol Imaging. (2023) 50:2830–45. doi: 10.1007/s00259-023-06255-8 37246997 PMC10317889

[9] Yadav S Kim ST Tuchayi AM Jiang F Morley A Saelee R . Comparison of 18F-DCFPyL and 68Ga-PSMA-11 for 177Lu-PSMA-617 therapy patient selection. Front Oncol. (2024) 14:1382582. doi: 10.3389/fonc.2024.1382582 38993644 PMC11238039

[10] Swiha M Papa N Sabahi Z Ayati N John N Pathmanandavel S . Development of a visually calculated SUVmean (HIT Score) on screening PSMA PET/CT to predict treatment response to 177Lu-PSMA therapy: Comparison with quantitative SUVmean and patient outcomes. J Nucl Med. (2024) 65:904–8. doi: 10.2967/jnumed.123.267014 38637137

[11] Kuo PH Morris MJ Hesterman J Kendi AT Rahbar K Wei XX . Quantitative 68Ga-PSMA-11 PET and clinical outcomes in metastatic castration-resistant prostate cancer following 177Lu-PSMA-617 (VISION Trial). Radiology. (2024) 312:e233460. doi: 10.1148/radiol.233460 39162634 PMC11366674

[12] Chen DC Buteau JP Papa N Akhurst T Alipour R Bollampally N . Prognostic value of initial imaging and PSA change with [177Lu]Lu-PSMA-617 radiopharmaceutical therapy in patients with metastatic castration-resistant prostate cancer: A ProsTIC Registry analysis. J Nucl Med. (2025) 66:1914–20. doi: 10.2967/jnumed.125.270804 41067860

[13] Karpinski MJ Civan C Rauscher I Güven O Eiber M Hoberück S . New prostate cancer risk groups by PSMA-PET (PPP3): An international, retrospective, registry-based cohort study. Lancet Oncol. (2026) 27(4):480–90. doi: 10.1016/S1470-2045(26)00016-1 41819113

