# Supplementary material for: Comparative analysis of treatment response to PSMA radioligand therapy using Ga-68 PSMA-11 and F-18 DCFPyL PET imaging
Source: Front Oncol. 2026 Oct 2;16:1932583. doi: 10.3389/fonc.2026.1932583 (PMC13672183; doi:10.3389/fonc.2026.1932583)
Supplement: Supplementary file 1 [file SupplementaryFile1.docx]

**Supplementary Table 1. Multivariable sensitivity analyses restricted to patients with a PET-to-RLT interval ≤90 days**

| **Variable** | **PSA50 response OR (95% CI)** | **P value** | **Overall survival HR (95% CI)** | **P value** |
| --- | --- | --- | --- | --- |
| Age at first RLT | 1.034 (0.980–1.092) | 0.225 | 0.987 (0.959–1.017) | 0.396 |
| Baseline PSA | 1.000 (0.999–1.001) | 0.995 | 1.000 (1.000–1.000) | 0.916 |
| PET tracer (DCFPyL) | 1.296 (0.533–3.150) | 0.567 | 0.856 (0.528–1.388) | 0.529 |
| SUVmean | 1.260 (1.084–1.464) | 0.003 | 0.891 (0.817–0.971) | 0.008 |
| TTV (log-transformed) | 1.304 (0.929–1.830) | 0.125 | 1.381 (1.139–1.675) | 0.001 |

The sensitivity analysis included 112 patients who initiated RLT within 90 days of baseline PSMA PET. TTV was natural log-transformed for regression analyses. OR, odds ratio; HR, hazard ratio; CI, confidence interval; PSA, prostate-specific antigen; TTV, total tumor volume; RLT, radioligand therapy.

**Supplementary Table 2.** Characteristics of the previously published cohort

|  | **Total**  **(n=80)** | **Ga-68 PSMA-11 (n=47)** | **F-18 DCFPyL**  **(n=33)** | **p-value** |
| --- | --- | --- | --- | --- |
| **Age at first RLT**  **(median [IQR])** | 72 [67, 79.25] | 74 [68, 80] | 74 [68, 80] | 0.36 |
| **Prior treatment** |  |  |  |  |
| **Androgen deprivation treatment** | 80 (100%) | 47 (100%) | 33 (100%) | 1.00 |
| **Androgen receptor pathway inhibitor** | 80 (100%) | 47 (100%) | 33 (100%) | 1.00 |
| **Chemotherapy** | 79 (99%) | 46 (98%) | 33 (100%) | 1.00 |
| **Site of disease on PSMA PET** |  |  |  |  |
| **Prostatic fossa** | 23 (29%) | 14 (30%) | 9 (27%) | 1.00 |
| **Lymph nodes** | 57 (71%) | 33 (70%) | 24 (73%) | 1.00 |
| **Bone** | 77 (96%) | 46 (98%) | 31 (94%) | 0.57 |
| **Viscera** | 29 (38%) | 18 (38%) | 11 (33%) | 0.81 |
| **SUV_max_ (median [IQR])** | 46.65 [29.91, 79.60] | 39.32 [25.97, 75.61] | 55.26 [36.57, 89.67] | 0.05 |
| **SUV_mean_ (median [IQR])** | 7.98 [5.59, 11.33] | 7.56 [5.46, 11.28] | 8.40 [6.18, 11.74] | 0.39 |
| **Total Tumor Volume (mL)** | 605.63 [189.12, 1268.06] | 452.48 [98.73, 1144.06] | 734.81 [426.57, 1270.78] | 0.14 |
| **Days from baseline PSMA PET to the 1st cycle (median [IQR])** | 67 [42, 95] | 72 [54, 106] | 64 [41, 77] | 0.26 |
| **Number of Cycles**  **(median [IQR])** | 4 [2, 6] | 4 [2, 5] | 4 [3, 6] | 0.25 |
| **Baseline PSA**  **(median, ng/mL [IQR])** | 134.45 [37.14, 465.36] | 134.45 [42.58, 300.5] | 209.18 [36.76, 1307.99] | 0.22 |
| **PSA50 Response rate** | 44 (55%) | 20 (43%) | 24 (72%) | 0.03 |

**Supplementary Table 3. Univariable and multivariable analyses for PSA50 response and overall survival in the previously published cohort**

| **Logistic regression analysis for PSA50 response** | | | | | | | |
| --- | --- | --- | --- | --- | --- | --- | --- |
|  | **Univariable** | | | **Multivariate** | | | |
|  | OR | 95% CI | *P value* | OR | 95% CI | *P value* | |
| Age at first RLT | 1.021 | 0.966–1.081 | 0.458 | 1.035 | 0.971–1.105 | 0.291 | |
| Baseline PSA | 1.000 | 1.000–1.001 | 0.363 | 1.000 | 1.000–1.001 | 0.514 | |
| PET tracer (DCFPyL) | 3.049 | 1.052–8.839 | 0.040 | 2.816 | 0.901–8.800 | 0.075 | |
| SUV_max_ | 1.009 | 0.996–1.022 | 0.164 |  |  |  | |
| SUV_mean_ | 1.193 | 1.017–1.399 | 0.030 | 1.210 | 1.021–1.433 | 0.028 | |
| TTV  (log-transformed) | 1.299 | 0.939–1.795 | 0.114 | 1.246 | 0.828–1.874 | 0.291 | |
| **Cox proportional hazard regression for overall survival** | | | | | | |  |
|  | **Univariable** | | | **Multivariate** | | | |
|  | HR | 95% CI | *P value* | HR | 95% CI | *P value* | |
| Age at first RLT | 0.990 | 0.960–1.021 | 0.528 | 0.986 | 0.952–1.021 | 0.422 | |
| Baseline PSA | 1.000 | 1.000–1.000 | 0.004 | 1.000 | 1.000–1.000 | 0.075 | |
| PET tracer (DCFPyL) | 0.873 | 0.523–1.457 | 0.603 | 0.850 | 0.496–1.458 | 0.556 | |
| SUV_max_ | 0.994 | 0.987–1.000 | 0.062 |  |  |  | |
| SUV_mean_ | 0.933 | 0.872–0.999 | 0.046 | 0.942 | 0.877–1.012 | 0.104 | |
| TTV  (log-transformed) | 1.243 | 1.009–1.529 | 0.040 | 1.176 | 0.948–1.460 | 0.140 | |
